# Pediatric dacryolith masquerading as congenital nasolacrimal duct obstruction

**DOI:** 10.1016/j.ajoc.2024.102117

**Published:** 2024-07-20

**Authors:** Upasana Pokal, Ashish Ranjan, Mohammad Javed Ali

**Affiliations:** Govindram Seksaria Institute of Dacryology, L.V. Prasad Eye Institute, Hyderabad, 34, India

**Keywords:** Dacryolith, Lacrimal sac, Lacrimal, Congenital nasolacrimal duct obstruction, Congenital

## Abstract

**Purpose:**

To report a rare case of a pediatric dacryolith masquerading as congenital nasolacrimal duct obstruction (CNLDO).

**Observations:**

A two-year-old male child presented with history of intermittent epiphora and discharge since the age of six months. Clinical evaluation demonstrated raised tear meniscus height and delayed fluorescein dye disappearance test in the right eye. Lacrimal irrigation of the right eye under general anesthesia demonstrated 90 % regurgitation (subjectively) of mucoid fluid with a hard stop. Nasal endoscopy examination demonstrated a dacryolith obstructing the opening of the nasolacrimal duct (NLD) in the inferior meatus. The dacryolith was teased out of the NLD and following its removal the lacrimal irrigation was freely patent. At six-months post operative follow up, epiphora resolved and the child was asymptomatic.

**Conclusions and importance:**

While cases of canaliculitis is uncommon in pediatric age group, it is rare to find a NLD dacryolith in a toddler. To the best of the authors’ knowledge, there are few prior reports on pediatric NLD dacryolith masquerading as CNLDO in a toddler (1–3 years).

## 1 Introduction

Dacryolithiasis is a relatively common condition and has been reported as an incidental finding in 5.7%–18 % during the dacryocystorhinostomy surgery.^1^ The etiopathogenesis of dacryolithiasis has recently been decoded.^2^ It is usually reported in the 6th decade of life with the mean age at presentation being 52.5 years (range: 22–77 years).^1^^,^^3^ While cases of canaliculitis is uncommon in pediatric age group, it is rare to find a nasolacrimal duct (NLD) dacryolith in a toddler.^4^ To the best of the authors’ knowledge, there are only few reports on pediatric NLD dacryolith masquerading as congenital nasolacrimal duct obstruction (CNLDO).^5^^,^^6^ The present case reports a 2-year-old child referred with a diagnosis of CNLDO where endoscopy examination demonstrated a large dacryolith obstructing the NLD opening in the inferior meatus.

## 2 Case report

**Fig. 1:**
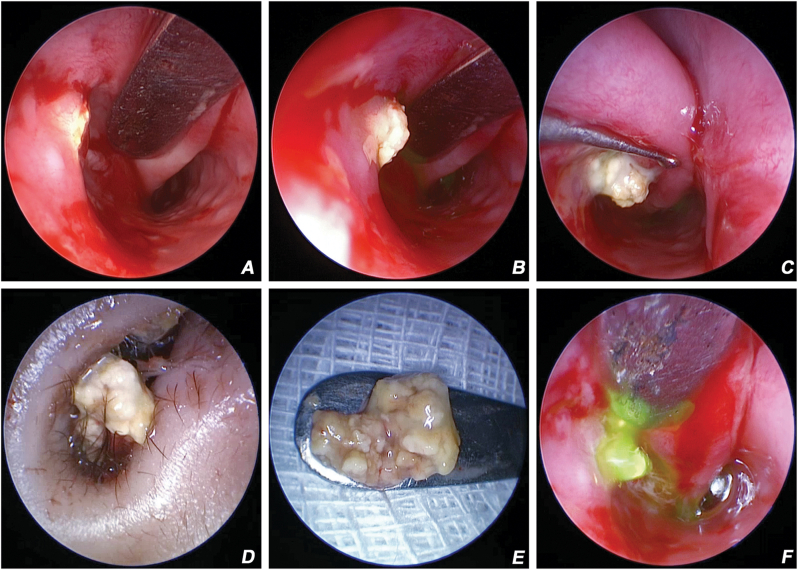
Endoscopic image of the right inferior meatus demonstrating the dacryolith obstructing the opening of the nasolacrimal duct (NLD) (Fig. 1A). Note the increasing projection of dacryolith beyond the opening upon pressure irrigation (Fig. 1B), and retrieval of the dacryolith with Freer's elevator (Fig. 1C). External image demonstrating retrieval of the dacryolith from the nasal vestibule (Fig. 1D). Gross examination of the dacryolith showing the wider proximal segment (housed in the lacrimal sac) and the distal narrower segment of the NLD (Fig. 1E). Endoscopy image of the right inferior meatus demonstrating free flow of fluorescein dye from the NLD opening upon irrigation (Fig. 1F).

The report adhered to the Tenets of the Declaration of Helsinki. A 2-year-old male child was referred to the Dacryology services with a diagnosis of CNLDO. The parents reported right eye intermittent epiphora since the age of 6 months. Epiphora was associated with occasional discharge and symptomatology was of persistent nature from the age of 18 months. There was no history of acute dacryocystitis or any surgical intervention. Clinical evaluation demonstrated raised tear meniscus height and delayed fluorescein dye disappearance test in the right eye. The left eye was normal. An impression of right eye CNLDO was made, and the patient was advised for a probing under nasal endoscopy-guidance. Lacrimal irrigation of the right eye under general anesthesia demonstrated 90 % regurgitation (subjectively) of mucoid fluid with a hard stop. Nasal endoscopy examination demonstrated a dacryolith obstructing the opening of the NLD in the inferior meatus (Fig. 1A). Repeat irrigation pushed part of the dacryolith beyond the opening of the NLD (Fig. 1B). The dacryolith was gently maneuvered out from the NLD into the inferior meatus and removed from the nasal cavity with a downward massage stroke of the Freer periosteum elevator (Fig. 1C and D). Gross examination demonstrated a soft dacryolith taking the shape of the distal lacrimal drainage (Fig. 1E). It had a broad proximal segment (segment in the lacrimal sac) and distal narrow segment (one in the NLD) (Fig. 1E). Histopathological and microbiological examination was not performed. Lacrimal irrigation following dacryolith removal was freely patent (Fig. 1F). The post-operative period was uneventful. At six months follow-up, epiphora resolved and the child was asymptomatic.

## 3 Discussion

Dacryolith refers to concretions formed within the lacrimal drainage system and can be infectious or non-infectious. Infectious dacryoliths are commonly noted within the canaliculi and are also referred to as canaliculoliths.^1^ Non-infectious dacryoliths usually originate from the lacrimal sac and the NLD and are predominantly composed of mucopeptides and hence also referred to as mucopeptide concretions. When unspecified, dacryolith refers to concretions within the lacrimal sac and the NLD.^2^ The common presentation documented in adults includes epiphora, discharge, dilated lacrimal sac, and acute dacryocystitic retention.^1^ However, the symptomatology in pediatric patients is not documented owing to the rarity of the presentation.

The utility and advantages of endoscopic guidance in complex CNLDO is now well established.^7^ The present case is yet another example where the surgeon would have likely missed the diagnosis of a dacryolith obstructing the opening of the NLD in the inferior meatus. Besides, the management would have been suboptimal in the absence of endoscopy. Under direct nasal endoscopic guidance, the dacryolith can be manoeuvred out of the NLD in a controlled and atraumatic manner.

In summary, pediatric NLD dacryolith presenting as CNLDO is a rarely reported lacrimal drainage disorder, although its incidence may be higher if routine endoscopic examination was carried out in these patients.^8^ The routine use of nasal endoscopy is controversial with strong arguments on both sides of the divide.^7^^,^^9^ The approach can be surgically challenging and endoscopy guidance proved useful in the diagnosis and management of a pediatric NLD dacryolith.

## Patient consent

Consent to publish this case report has been obtained from the patient(s) in writing. This report does not contain any personal identifying information.

## Funding

Hyderabad Eye Research Foundation.

## Authorship

All authors attest that they meet the current ICMJE criteria for Authorship.

## Financial disclosure

Prof. Mohammad Javed Ali receives royalties from Springer for the textbook “Principles and Practice of Lacrimal Surgery” and for the treatise “Atlas of Lacrimal Drainage Disorders.”

## Funding

10.13039/501100005809Hyderabad Eye Research Foundation and J C Bose Fellowship grant of the 10.13039/501100001843Science and Engineering Research Board, India.

## CRediT authorship contribution statement

**Upasana Pokal:** Writing – original draft, Methodology, Investigation, Data curation. **Ashish Ranjan:** Writing – original draft, Methodology, Investigation, Data curation. **Mohammad Javed Ali:** Writing – review & editing, Writing – original draft, Supervision, Conceptualization.

## Declaration of competing interest

The authors declare that they have no known competing financial interests or personal relationships that could have appeared to influence the work reported in this paper.
